# Social media and climate-related disaster management in Africa: A force-field analysis

**DOI:** 10.4102/jamba.v17i1.1753

**Published:** 2025-04-04

**Authors:** Agwu A. Ejem, Somtochukwu V. Okeke, Rachael O. Ojeka-John, Emmanuel T. Adekeye

**Affiliations:** 1 Department of Mass Communication, Faculty of Business and Social Sciences, Landmark University, Omu-Aran, Nigeria; 2 Department of Mass Communication, Faculty of Arts, University of Nigeria, Nsukka, Nigeria

**Keywords:** climate change, climate-related disasters, disaster management, force field analysis, risk communication, risk reduction, SDG 13, SDG 11, social media

## Abstract

This article reviewed bodies of existing local and international literature to provide multi-level insights into Africa’s readiness to standardise the adoption of social media and associated technologies in managing the numerous climate-related disasters in Africa, including storms, floods and droughts. Social media is making serious inroads in disaster management globally, except in Africa, with countries such as the United States of America, Japan, Haiti, Australia and so on, effectively deploying social media technologies in different cycles of disaster management, particularly since 2010. To encourage disaster management stakeholders in Africa to mainstream the involvement of social media in disaster management, this study examined Africa’s prospects using force-field analysis that assessed the social, financial, policy, technological and other factors that inspire or restrain the effective and comprehensive adoption of social media technologies in disaster management. The force-field analysis demonstrated that disaster management stakeholders in Africa have all the tools and conditions to adopt social media technologies in climate-related disaster management on the continent.

**Contribution:**

Driving forces such as the steady Internet access and penetration in Africa, fast-growing social media penetration and adoption of mobile technology, Africa having four of the top 10 countries that spend the most time on social media globally, growing investments in Internet infrastructure and communalistic nature of African societies, among others, are pointers of Africa’s readiness to mainstream social media technologies in climate change-related disaster management.

## Introduction

The world has experienced deadly climate-related disasters in the past decade, and a report by the World Health Organization (2024) showed that Africa accounted for 59.6% of the total population affected by disasters globally in 2022, which is a significant increase from a global average of 9.3% between 2002 and 2021. Africa is susceptible to climate-related disasters, including storms, floods and droughts. Floods and droughts have had some of the worst impacts on the continent, in terms of loss of lives, property, landmarks and livelihoods and the spread of diseases (Ejem et al. 2023; Molua, Mendelsohn & Akamin 2020). Most recently, there has been an unprecedented wave of climatic and geophysical disasters on the continent. In the last decade, several West African countries have experienced some of the worst floods in recorded history, resulting in devastating impacts (Brempong et al. 2023), for instance, between 2011 and 2020, there were 1187 deaths linked to flooding in Nigeria, and in 2022 alone, the country experienced the worst flooding in a decade with 603 persons dead, more than 2400 persons injured and about 1.5 million people displaced (Luu, Von Meding & Mojtahedi 2019; Oguntola 2022; Umar & Gray 2022); Ghana recorded 49 natural disasters, comprising floods and storms between 2011 and 2020 (Sasu 2022); East African countries lost at least 200 lives and have had tens of thousands displaced and livelihoods lost because of floods and landslides (Hairsine 2024); southern African countries suffered a prolonged drought that has seriously threatened food security in the region; in Southeastern Africa, Malawi alone has suffered 16 flooding events since 2010 and heatwaves are leading to deadly wildfires and very distressing impacts in North Africa (Amosi & Anyah 2024). These are only a few cases, and climate change is increasing in frequency and severity. According to a Press Release by the African Union (2023), in 2022 alone, the economic toll of climatic and geophysical hazards in the continent amounted to 38.5 billion US dollars, surpassing the combined gross domestic products (GDPs) of 15 African countries.

Managing disasters involves collaborations among stakeholders such as the government, the public sector, voluntary organisations, businesses and individuals. These stakeholders are a combination of those affected by disaster events and those responsible for their mitigation, and to be able to coordinate these groups, effective communication is necessary. To execute effective communication, it is vital to consider the use of technologies that broadly overcome the barriers of traditional media, especially limited reach, sensory appeal and passivity. These align with the characteristics of social media and its associated technologies.

Social media is a form of media that facilitates social interaction and communication using online Internet-based platforms such as social networks and blogs, bookmarking sites, collaborative projects, content communities and social reviews (Tuten 2023). Leveraging its characteristics of collectivity, clarity, competencies and collaborations, social media is now a widespread media category for online discourse, allowing users to create content, share it, bookmark it as well as network. These characteristics make social media an important platform for information gathering and dissemination, collaborative problem-solving and decision-making and disaster preparation and training.

Recently, government and non-governmental organisations in many parts of the world have effectively leveraged the characteristics of social media to strengthen their capabilities to manage disasters, specifically in disaster preparation, disaster response, disaster recovery and disaster mitigation. For instance, a social media tool known as Ushahidi was used for disaster mapping and crowdsourcing during the 2010 Haiti Earthquake (Heinzelman & Waters 2010), and another one known as Sahana was developed and used by the New York City’s Office of Emergency Management for Open Source disaster management (Duc, Vu & Ban 2014). There are many more cases of social media being used by government and non-governmental organisations in disaster management.

Therefore, our position in this article is that, with the growing climate-change concerns in Africa and the rise of climate-related disaster events and impacts on the continent, Africa should seriously consider leveraging the advantages of social media technologies to mainstream their adoption in managing disaster events on the continent and help mitigate the effects of climate change-related disasters. In this article, we assessed the continent’s prospects of adapting these technologies to manage climate-related disasters by evaluating the forces that drive their effective implementation and those that restrain it. Drawing attention to these forces is expected to help those responsible for climate-related disaster mitigation to take advantage of the driving forces while weakening or becoming aware of and knowing how to navigate the restraining forces.

## Climate-related disaster management

Disaster is a serious interruption of the functioning of a community or society, involving prevalent human, material, environmental and economic impacts that exceed the capability of the affected community or society to cope with their limited resources (Al-Husain 2023). Climate-related disasters are naturally occurring disasters that are caused or heightened by the impacts of climate change, such as geophysical (landslides, earthquakes, Tsunamis, etc.), hydrological (floods and avalanches), climatological (droughts, extreme temperatures and wildfires) and meteorological (storms, waves, storm surges and cyclones)

While it is believed in the literature that disasters cannot be completely stopped, it is possible to manage them by mitigating their human, economic, material or environmental impacts. Researchers agree that the objectives of managing disasters are to prepare for an impending disaster event, reduce or avoid potential loss to hazards (Wilkinson, Pforr & Weingärtner 2020), organise and direct resources to deal with a disaster event (Okyere et al. 2024), guarantee rapid and appropriate help to victims when needed, achieve quick and lasting recovery (Farahani et al. 2020), direct the roles of stakeholders such as responders, public and public sector organisations, communities, nongovernmental organisations, volunteers, faith-based organisations and so on (Ejem & Ben-Enukora 2023; Okyere et al. 2024).

Disaster management involves effectively preparing for and responding to disasters and creating the framework to mitigate or reduce vulnerability to hazards and cope with disaster. According to Sawalha (2023), disaster management is ‘the strategic organisation and management of resources and responsibilities for handling the humanitarian aspects of emergencies, specifically preparation, response and recovery to reduce the impact of disasters’. To manage disasters, potential calamities associated with hazardous situations are anticipated and addressed strategically and effectively.

Disaster management does not only involve the top-down all-hazard approach of official coordination of disaster mitigation, preparation, response and recovery. With the increasing threat of disasters every year as a result of climate change and unpredictable weather patterns, disaster management also involves the inclusive participation and representation of the local population to build communities that are sustainable and resilient to disaster events (Morelli et al. 2021). These communities are equipped, sometimes with help from official sources, to reduce the risk of hazards, cope with disasters if they occur and minimise their impact on their livelihoods and health (Fauziyanti & Hizbaron 2020).

The five stages of the disaster management cycle include prevention, which involves identifying potential hazards and finding ways to mitigate their impact; mitigation, which consists of reducing the loss from disasters using structural and non-structural measures, and mediating future threats; preparedness, which involves plan and train individuals, communities, businesses and organisation on roles and responsibilities in the event of a disaster; response, which involves short-term and long-term activities that follow the occurrence of the disaster, including removal of any ongoing hazards and recovery, which affects to stabilising the affected communities or areas and the restoration of all essential community functions (Shah et al. 2023).

## Social media penetration in Africa

Social media include all the technologies and platforms that enable users universally to create and share information in virtual space, allowing for spontaneous two-way and many-to-many dialogue in the online environment. These technologies rely on collaborative, decentralised and community-driven peer-to-peer networks (Dufty 2015) and make users, not just content consumers but also active content creators. The platforms include social networks, blogs, bookmarking sites, collaborative projects, chat rooms, content communities, apps, discussion forums, crowdsourcing platforms and social reviews. The most popular social networking platforms are Facebook, X, WhatsApp, LinkedIn, Instagram, YouTube and MySpace, among others. Records show that one out of four persons in the world regularly use some forms of social media.

In Africa, records showed that countries in the Northern and Southern sub-regions have the largest share of social media users in Africa, with some accounts saying 56% and 45% of the population, respectively (Bhanye, Shayamunda & Tavirai 2023) and another account saying 40.4% and 41.6% of the population, respectively (Statista 2024). However, Nigeria tops the list of countries with the most social media (and Internet) penetration in Africa, followed by Egypt and South Africa. In contrast, only about 10% of the populace in Central Africa uses social media, making it the lowest in Africa and the lowest globally in terms of regional share.

Facebook dominates the African social media market in terms of market share, generating over 50% of the social media traffic. This is followed by YouTube and X (formerly known as Twitter). As of 2022, there were 271 million Facebook subscribers in Africa, but the figure is expected to reach 377 million by 2025. Also, in 2022, YouTube and *X* had 180 million and 24 million users, respectively. Records also showed that there were more male (about a share of 60%) social media users in Africa. Out of the top 10 countries that spend the most time on social media in the world, four of them are in Africa. Nigeria tops the global list with 4 h and 20 min spent on social media daily, South Africa is 3rd (3 h:44 min), Ghana is 6th (3 h:23 min) and Kenya is 7th (3 h:22 min) (Business Insider Africa 2023).

However, the bigger picture is that Internet – and social media – penetration is fast growing in Africa, especially with increasing Internet use on the continent, which is driven by the widespread adoption of mobile technology and huge investments in Internet infrastructure. As of 2022, Statista reported that there are approximately 570 million Internet users in Africa.

Nevertheless, Internet connectivity is lower in Africa than in other regions of the world. The optical fibre footprint in the continent is limited, and there are countries in the continent that lack the necessary high-speed telecommunications infrastructure. More so, there are more fixed broadband access rates in the world which cost a lot of money, far more than the average recommended by the International Telecommunication Union (ITU).

## Use of social media technologies in disaster management

Social media has become a popular global means of creating, sharing and interacting with user-generated content in a collaborative and participatory way (Young et al. 2020) at all times, including during disaster events. Social media has been used for disaster risk warning and to coordinate response and recovery. Throughout the disaster management cycle, social media can be used for disaster forecasting, preparation and training, information gathering and dissemination, collaborative problem-solving and decision-making.

People use social media in disaster management as a suitable medium for providing fantasy escape and relief from stress during horrendous times, determining disaster magnitude, self-mobilise, checking in with people in their networks, maintaining a sense of community and seeking emotional support (Fraustino, Liu & Jin 2012), address rumours and mobilise volunteers and organisations. More so, people turn to social media to seek timely and unfiltered information during disaster events. Social media disaster mapping is useful in providing relief and humanitarian rescue. Disaster managers mine through databases of tagged content on social media and social review sites to identify relevant concerns and themes being expressed online.

Those in charge of mitigating disasters such as responders, public sector agencies, private sector organisations, nongovernmental organisations, volunteers, faith-based organisations and so on take advantage of the opportunities in social media to manage disaster across all stages of the disaster life cycle in the following ways.

During the mitigation stage, stakeholders engage social media to share hazard prevention and reduction strategies such as the building of fences, clearing of dams, drainages, waterways, etc. (Lam et al. 2023; Seneviratne et al. 2023). The local populations, in a bid to build sustainable and resilient communities in anticipation of disasters, use social media to encourage members to engage in disaster mitigation activities such as practising dry season farming in usually flooded areas, clearing dams, drainages and waterways, planting trees, building local walls with sandbags and other Indigenous disaster prevention strategies (Auliagisni, Wilkinson & Elkharboutly 2022; Toyoda et al. 2021). These collaborative, inclusive and local actions by the local communities have been known to reduce disaster risks and mitigate losses (Toyoda et al. 2021)

During the preparation stage, social media is used to incorporate early warning systems for timely alerts, raise awareness among the populations about impending disasters (Al-Wathinani et al. 2023), facilitate discussions about disaster preparedness and provide information about shelter, aids, medicine and other supplies (Cvetković, Nikolic & Ivanov 2023; Okocha, Agbele & Kente 2023; Zamarreño-Aramendia et al. 2020). Communities also collaborate and use social media to share preparation activities such as locations, evacuation plans, safe routes and coping strategies (Lam et al. 2023, Seneviratne et al. 2023). Social media has also been used for disaster susceptibility mapping (Li et al. 2023).

During the disaster response stage, social media ensures that real-time information about the disaster is shared by stakeholders. Social media is used in search, rescue and recovery efforts and to mobilise donations. Communities use social media to stay connected, share resources, ask for and provide help to members and share updates (Seneviratne et al. 2023). The government uses social media to provide real time weather alerts, information about shelters and safety tips to at-risk populations. Artificial intelligence has been integrated into social media to create data-driven solutions during disaster preparation and response (Jin, Yang & Fang 2020). Social media is used to capture real-time experiences of populations affected by disaster (Moghadas et al. 2023) and to reduce response time (Aboualola et al. 2023), among others.

Also, social media creates avenues for collaboration during the response stage. Kuligowski et al. (2019) outlined how emergency managers and other stakeholders, including volunteers, band together to form global social media communities of practice known as Virtual Operations Support Teams (VOSTs) to share ideas during disasters. These teams lend support to the overwhelmed on-site responders.

During disaster recovery, social media helps to alert stakeholders, relief organisations and the public about the specific needs of communities affected by disasters. It can also help to track and assess disaster damage impact in readiness for rebuilding (Ogie et al. 2022). Social media is effective in canvasing for donations and financial support after a disaster, providing support for the socioeconomic and physical well-being of victims, providing mental and emotional support, information support, revitalising the business and economic activities of people affected by the disasters and mobilise post-disaster reconstruction and infrastructure services (Houston et al. 2015; Ogie et al. 2022)

Social media developers have helped in disaster response by integrating features that boost the use of the platforms during disaster events. For instance, the support features on Facebook that encourage the use of Facebook during disasters. Those features include Community Help, Safety Checks, Crisis Response and Fundraisers. Similarly, YouTube enables users to share complex information and upload and share disaster-related videos that capture the real-time actual experiences of disaster-affected people and communities. Also, image-based social media platforms such as Pinterest, Instagram, Flickr, TikTok, Tumblr, 500px, etc. can help disaster managers track the impact of disaster damage and the recovery process. More so, mobile applications have been used by disaster managers to provide at-risk populations with real-time safety tips, National Weather Service (NWS) alerts and locations of operational shelters (Federal Emergency Management Agency [FEMA] 2019). Online discussion platforms and news aggregators are used to spread, collect and analyse information more effectively and comprehensively.

### Case studies

Social media has been extensively used in disaster management in Haiti, Australia, America, New Zealand and the Philippines as follows:

#### New Zealand

New Zealand has used Ushahidi, an open-source crowd-sourcing platform developed by Kenyans, for crisis mapping, most notably during the 2011 Christchurch Earthquake. The platform is used to aggregate and graphically display actionable social media content and official messages. Similarly, New Zealand uses Google Person Finder to collect and share information about missing persons during disasters (Los Angeles Times 2011). More so, they use dedicated *X* and Facebook pages to distribute critical information and meet the humanitarian needs of the community (Homeland Security 2013).

#### Philippines

The Philippines uses a single hashtag on *X* to easily access emergency information during a disaster to help the processes of monitoring, tracking and consolidating information, before, during and after a disaster event (Ludwig et al. 2015). They have also developed Agos to facilitate information processing through social media and MicroMappers, which enables disaster managers to have more situational awareness to be able to allocate resources and coordinate logistics during disaster events. They also use OpenStreetMap for disaster mapping (Murwani, Wulandari & Putri 2018).

The country also uses PhilAWARE, an innovative social media technology designed for monitoring new hazards, early warning and decision-making. Other objectives of the system are to increase disaster resilience, reduce loss of lives and properties and facilitate greater cooperation before, during and after emergencies. MapaKalamidad.ph was also launched to support the PhilAWARE in disaster mitigation, preparation, response and recovery (Pacific Disaster Center 2021).

#### Haiti

Social media is used for crisis mapping in Haiti, most notably during the 2010 Haiti Earthquake (Heinzelman & Waters 2010). Ushahidi, the open-source interactive mapping solution that is becoming a global system, is used to geotag *X* messages and other mappable content from other new media platforms in Haiti. Users rely on the map to zoom into an area and read the associated content, and this availed valuable information for bodies that provided response and recovery services (Homeland Security 2023). On the other hand, the American Red Cross used new media technologies for charity campaigns that enabled them to raise money for Haiti relief during the 2010 Haiti Earthquake (Mavrodieva & Shaw 2021).

#### United States of America

Social media has influenced effective disaster management systems in the United States of America in many ways. There is always a coordinated and relentless distribution of information using social media and distribution of relief materials using triages extracted from social media messages. During the 2017 Hurricane Harvey, which was the joint-costliest tropical cyclone with 2005 Hurricane Katrina, causing $125 billion in damages, 103 fatalities and more than 30 000 displacements (Blake & Zelinsky 2018; Zou et al. 2023), social media was an alternative source of communication because of overload of the Houston 911 system (Hazarika et al. 2020; Rhodan 2017). Accounts like @HarveyRescue collected the addresses of people who needed assistance and shared their details publicly. Hashtags like #sosHarvey were used to flag citizen-victims on social media platforms. The neuro-net toponym recognition (NeuroTPR) was eventually developed to extract locations from social media rescue messages (Zou et al. 2023).

Social media was also extensively used for monitoring and collaborative communication by government agencies, non-governmental organisations, the news media and the public during Hurricane Isaac 2012 in the City of New Orleans and the Gulf Coast. The hashtags #Isaac and #NOLA were widely used to share information on various social media networks (Hazarika et al. 2020; Homeland Security 2023).

Also, a social media crowd-souring tool known as Sahana was developed and is used by the New York City’s Office of Emergency Management for Open Source disaster management. It is used to reduce the impact of disasters by tracking local needs coordinating across agencies and planning and managing evacuation (Duc et al. 2014). More so, the Homeland Security and Emergency Department always has dedicated social media accounts for disaster mitigation, preparation, response and recovery such as emergency blog, *X*, Facebook, YouTube, Flickr and paper.li (Homeland Security 2023).

In the United States of America, an application called *Harmany* is used to connect people who can provide operational shelter during disasters with those in need of them (Young et al. 2020). *Zello* is an application used in search, rescue and recovery efforts (Smith et al. 2018). *Nextdoor* is used by communities to allow neighbours to connect during a disaster, as well as share resources, distribute information, ask for help and share updates (Rab 2015).

#### Australia

Emergency services of Australia have expansively updated their emergency communications strategies by using many social media platforms to share timely information and encourage the public to engage and upload content to the news and media website of the Country Fire Authority (CFA). The website provides news, multimedia and a discussion board known as the CFA Forum (Homeland Security 2023; Smeaton & Davis 2014; Steinmetz et al. 2021).

The CFA has a robust social media policy for disaster management setup up and responsibilities (Smeaton & Davis 2014). They use *X* primarily as a distribution mechanism during active emergencies and Facebook as a platform to sustain more detailed dialogue during emergencies. The Australian government also funded an independent social media research, and the results were incorporated into a YouTube video to demonstrate the use of social media in spreading accurate and helpful alert messages during disasters (Homeland Security 2023). Australia also uses the Community Emergency Alert Network to generate important emergency alerts, notifications and updates. An interactive crisis map was launched during the 2010–2011 flooding in Victoria, and that was combined with the transmission of alerts and warnings through social media outlets.

#### Africa

While there is evidence that social media technologies have been used in some parts of Africa, they are not prevalent and mainstreamed into disaster management processes on the continent as they are done in our case studies. Social media has been used by government officials in response to incidents in parts of Africa in a limited capacity (Ben-Enukora et al. 2025; Saroj & Pal 2020), but the conclusions about the framework and their effectiveness in those limited situations are varied and imprecise. In Ghana, for instance, Kelly and Addo (2023) wrote that although global advances have been made in the use of social media technologies in disaster management, it remains ‘a contrary narration’ in Ghana, and, when they are used, the most prevalent method of adoption is merely posting disaster information on social media platforms. There is also evidence of limited use of social media and poor social media presence of disaster managers in Nigeria (Okocha et al. 2023), a lack of framework for effective use in disaster management in South Africa (Ramluckan 2016).

Ironically, the Ushahidi was created by a Kenyan lawyer and two tech corroborators primarily to counter election violence (Ajao 2017). However, it has found limited use in Africa and wide application in disaster management in Europe, America and Asia. Even when social media technologies are adopted into disaster management processes in Africa, there is no multicountry experience in the use of these technologies because of a lack of mainstreaming.

## Lewin’s force field analysis

Developed by Kurt Lewin in 1951, force field analysis provides a framework for assessing the factors that influence a situation, for instance, factors that inspire or restrain the effective and comprehensive use of social media in disaster management in Africa. Two contexts are looked at: the factors that drive the movement towards a goal and the factors that restrain the movement towards the goal (Mahmud, Mohd Nasri & Syed-Abdullah 2019). The essence of this analysis is to increase or take advantage of the critical driving force and decrease the critical restraining force. The driving factors inspire change or innovation, but the resisting forces pull the subjects or communities or organisation back to the current state. It has also been suggested that stakeholders need to focus on the driving forces as the restraining forces lead to negative outcomes (Mahmud et al. 2019). The rationale behind force field analysis is that after exploring the driving and restraining forces, the method should lead to a recommendation to either reinforce the forces for change or weaken the forces against change (Barry & Dick 2012). In the context of this research, the driving forces and restraining forces are inverted by nature; therefore, strengthening the driving forces will overcome the negative outcomes of the restraining forces.

**FIGURE 1:**
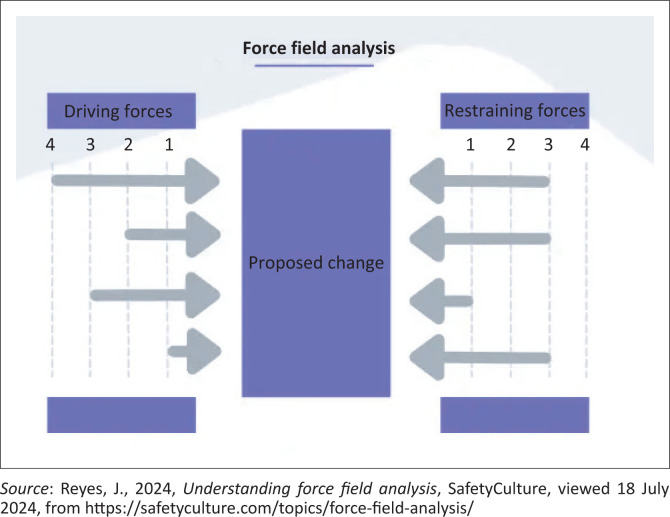
Framework for force field analysis.

Once the need for change or improvement is defined, the method is applied by putting corresponding values to the Force Field framework in Figure 1 and ranking them according to the extent to which they drive or restrain the movement towards the goal.

The advantages of the force field analysis are that it provides a visual representation of the factors that drive or restrain a situation, encourages a collaborative set-up in deciding the benefits and challenges of a planned change, permits a high-level outline of the situation by examining the factors and their implication to the proposed change and makes the evaluation process easier (Reyes 2024).

Force field analysis of the factors that encourage or restrain the effective adoption of social media in disaster management is important to enable disaster management stakeholders to be aware of and know how to take advantage of the driving forces and also be aware of and decrease the restraining forces. The ultimate aim is for disaster management stakeholders to effectively leverage the characteristics of social media technologies to strengthen their climate-related disaster management capabilities, specifically in the areas of disaster preparedness, disaster response, disaster recovery and disaster mitigation, in the same way, that it has been successfully adopted before, during and after disaster events in other continents.

## Driving and restraining forces in the adoption of social media technologies in climate-related disaster management in Africa

In ranking the driving and restraining forces in Table 1 and Table 2, they were weighted from 1 to 5 in terms of their relative impact on mainstreaming social media and associated technologies in disaster management in Africa. A value of 5 is most impactful and 1 is least impactful.

**TABLE 1:** Driving forces.

Driving forces	Rank
Steady Internet access and penetration in Africa (Faturoti 2022)	5
Social media penetration is fast growing (Adika 2023)	5
Four of the top 10 countries that spend the most time on social media globally are in Africa (Business Insider 2023)	4
Social media has been used by government officials in response to incidents (Kavota et al. 2020).	4
Adoption of mobile technology is growing (Ejemeyovwi and Osabuohien 2020)	5
Growing investments in Internet infrastructure (Ofori and Asongu 2021)	4
The most common social media activity is staying in touch with friends and family (Silver and Johnson 2018)	4
The African Union Cyber-security Convention framework (Elaiess 2017)	2
African societies are communalistic (Abakare and Okeke 2021)	4
Growth in social media activism in the continent (Sebeelo 2021)	2
Population explosion in Africa can be a driving force	3

**TABLE 2:** Restraining forces.

Restraining forces	Rank
Socio-economic issues affect equal access to the Internet and social media (Asongu and Odhiambo 2021)	4
Information-gathering activities on social media are less common than social activities (Silver and Johnson 2018)	2
Reduced capacity of governments and institutions to respond to disasters (Saroj and Pal 2020)	2
Social media is used by emergency officials in a limited capacity (Kavota, Kamdjoug and Wamba 2020)	2
Increasing ban on social media platforms in some African countries (Boateng 2022)	2
Information users in Africa have some of the lowest literacy levels (Akintolu and Uleanya 2021)	3
Internet connectivity is lower in Africa than in other regions (Tuten 2023)	3
Optical fibre footprint is limited (Mutegi 2016)	2
Broadband access is expensive (Ochoa et al. 2022)	4
Poor electricity supply and cost make social media use unstable (Sarkodie and Adams 2020)	5

## How do the driving forces indicate Africa’s readiness to adopt social media technologies in climate-related disaster management?

Based on the factors highlighted in Table 1, it is evident that Africa is ready for the full adoption of social media technologies in climate-related disaster management. As suggested in force-field literature, the focus of this discussion will be the driving forces as the restraining forces lead to negative outcomes (Mahmud et al. 2019).

Firstly, research has shown that there is a steady surge in Internet access and penetration in Africa, and it is leading to better social media penetration and more enhanced access to disaster-related information on social media (Adika 2023; Faturoti 2022). Many bodies of research evidence have stressed that a rise in Internet usage will lead to a transformation in how people work, communicate and access information (Akerele-Popoola et al. 2023; Ben-Enukora et al. 2022). Theoretically, this resonates with the assumptions of the theory of technological determinism, which posits that technology shapes perceptions and organises human experience and, as a result, causes inevitable change in society (Kellner 2021). In a nutshell, the growth of technology determines the development of social structure and cultural values. With this significant surge in Internet access and penetration in Africa, the continent is ready to effectively standardise the adoption of Internet technologies in disaster management, particularly in areas such as the sharing disaster prevention tips, provision of data sets, data exchange, creation of situational awareness, real-time data collection and analysis, search and rescue operations, communication and cooperation, monitoring of infrastructures, preparation and response and many more ways that the Internet has enabled in more technologically advanced societies.

Research has also found that tied with the increase in Internet access and penetration in Africa, there is a rise in the adoption of mobile technology (Ejemeyovwi & Osabuohien 2020) and social media penetration, meaning that more people have access to social media on the continent, and, as a result, more people have access to disaster-related information on social media (Robinson et al. 2020). During disasters, government agencies, non-profit originations and other stakeholders need platforms to communicate to citizens, deliver awareness and education messages and allow people to share experiences of risk and hazards, social media has become a veritable media to do that because of its capability to be interactive, collaborative and deliver timely and unfiltered information. However, evidence has shown that this can only effectively happen when there is reasonable social media penetration. Otherwise, the use of social media in disaster management will further widen the vulnerability of those who do not have access. Widespread social media penetration will ensure there is no information interruption in any geography where the disaster occurs (Okocha et al. 2023). As a result of all these developments in Africa, there is now reasonable access to real-time information, awareness and education about disasters, and more people are now able to share their experiences of risk and hazards during disaster events.

Having found that four of the top 10 countries that spend the most time on social media globally are in Africa (Business Insider 2023), we believe that social media is already a vital part of the life of the average African user. According to the schema theory, people make sense of the complex world based on their already-held experiences (Axelrod 1973). Africans already spend a lot of time on social media; so it will be a suitable medium to transmit, share and receive disaster-related information and education. They rely more on social media for information, communication and education, more than the mainstream media, with research showing that 57% of the people (2454 participants across the sub-regions in Africa) prefer social media, to TV (25%) and Radio (7%), especially among the younger people (Adika 2024). These also resonate with Gerbner’s cultivation theory, which stresses that the media has mainstreaming capabilities, especially among heavy users (Ejem, Ben-Enukora & Akanmode 2024).

Furthermore, the growing investments in Internet infrastructure have been responsible for improving Internet efficiency, creating more Internet platforms and reducing the cost of the Internet and social media use. As a result, Internet penetration is expected to continue to widen and social media use will only increase on the continent. Through private sector partnerships, local governments are investing in expanding broadband infrastructure in various parts of Africa (Nwokolo et al. 2023), and this is taking broadband networks to unserved and underserved areas (Gwaka, Haseki & Yoo 2023). Investments in Internet infrastructure have increased Internet usage in sub-Saharan Africa from 1% in 2000 to about 40% in 2023, and that is expected to significantly increase in the coming years. It is projected that by 2025, there will be 773 million Internet users in sub-Saharan Africa (Invest Africa 2023). Invest Africa (2023) also reported that while the World Bank maintains that the level of investments in broadband infrastructure currently is insufficient to meet the growing demand with $100 billion investments required, there is enough evidence to prove that there is steady growth in Internet infrastructure on the continent. Thus, ensuring that people in disaster-prone rural communities can afford smartphones, Internet access and social media connection.

Having reported an increase in social media activism on the continent (Sebeelo 2021), it is hoped that social media users will translate the same energy into creating and sharing disaster information during events. Social media have increased the capacity of communities in Africa to engage in social movements because of their ability to reach a much larger audience than the mainstream media. It has helped to mobilise and unite people in innovative ways. The success and spread of social media movements such as #EndSARS in Nigeria in 2020 is a pointer that social media can be effectively used on the continent to deliver awareness and education messages and allow people to share experiences of risk and hazards during disaster events. Similarly, there is evidence that social media has been used by government officials in response to incidents in parts of Africa, and they have shown to be effective even in those limited situations (Saroj & Pal 2020).

It is on record that social media have been deployed to manage disasters in places such as Mozambique, Malawi, Zimbabwe and Madagascar (Kalonde et al. 2023), and there is a growing academic interest in how it can be deployed in other parts of Africa (Kavota et al. 2020). The challenge, however, is that social media is used by emergency officials in a limited capacity (Kavota et al. 2020), and there is a lack of framework for its effective use (Ramluckan 2016). This lack of governance and framework is why a social media application developed in Kenya is rather finding great use in Europe, Asia and America (Ajao 2017). These limitations can be overcome if African countries develop the needed governance and framework for the extensive and effective use of social media in disaster management, and if disaster management stakeholders can take advantage of the growing Internet and social media penetration in Africa (Adika 2023; Faturoti 2022), and the fact that Africans spend good time on social media and rely on it for most of their information and connectivity (Business Insider 2023). The stakeholders should leverage that experience and the other driving forces to mainstream the adoption of social media in disaster management.

The evidence that the most common social media activity in Africa is staying in touch with friends and family is connected with the fact that African societies are communalistic (Abakare & Okeke 2021). As a result, safety is a community affair, and everyone in the community is traditionally concerned with the other persons in the community and their well-being. In Africa, our communities are traditionally organised to satisfy the basic needs of all the members; the people celebrate everything together, mourn together and share things. This culture prepares Africans to share disaster-related information with friends, family and members of the communities when there is a disaster event, and that can be facilitated using social media. This is a remarkable driving force in Africa because research has shown the role of communities in disaster prevention, mitigation, response and recovery (Ogie et al. 2022). To prevent the occurrence of new disasters or mitigate disaster risks, the local stakeholders engage social media to galvanise support for indigenous risk prevention or reduction strategies such as crowdfunding the clearing of drainages and dams, building sand-bag walls along shores, planting trees, etc. In preparing for disasters, communities collaborate and engage social media to share preparation activities such as locations, evacuation plans, safe routes and coping strategies (Lam et al. 2023; Seneviratne et al. 2023). In responding to disasters, communities use social media to stay connected, share resources, ask for and provide help to members and share updates. During the recovery stage, communal societies share resources, mostly through crowdsourcing via social media, to help victims get back on their feet after disasters (Ogie et al. 2022).

We also feel that population explosion in Africa can be a driving force as a larger population can boost human capital promises, which can in turn boost technological development (Ahmad, Idrus & Rijal 2023). Aside from having a large population, the majority of Africa’s population is made up of young people (Diao et al. 2019), and research has shown that a large and young population is an economic asset because of their aspirations, energy, technological savviness, innovativeness and creativity (Thafer 2023). The most popular social media platforms were founded by young entrepreneurs, and these media have become integral parts of disaster management in the world.

## Conclusion

There is enough evidence from the force field analysis to conclude that disaster managers in Africa have all the tools and conditions to leverage social media technologies in climate-related disaster management in the continent. The communalistic character of much of African society, the steady Internet and social media access and penetration, growing adoption of mobile technology and regular use of social media, among others, are indicative that communities, public sector agencies, private sector organisations, nongovernmental organisations, volunteers and faith-based organisations in Africa can effectively standardise the use of social media and associated technologies in disaster prevention, preparation, response and recovery. More so, the nature of the African society means that the continent can integrate both community-based and top-down approaches to disaster management using social media and associated technologies. While this has been applied in a limited capacity by some African countries, there is a need to mainstream the adoption of social media platforms, applications and frameworks in disaster management on the continent.

### Recommendations

Various governments in Africa should provide the needed governance and framework for the extensive and effective use of social media and associated technologies in disaster management. As Ramluckan (2016) suggested in South Africa, this will mitigate most of the factors that limit the standard adoption of social media and associated technologies in disaster management on the continent.

Moreover, there should be multicountry adoption of social media technologies in disaster management on the continent, in the same way, that Ushahidi, Sahana and other technologies have been effectively used in many countries in Asia, Europe and America, after trialling them in one country and finding them effective in disaster management.

Most of the restraining factors will be overcome with adequate investment in electricity, Internet and mobile infrastructure in Africa, as it will reduce the cost of broadband, improve optical fibre footprint, increase Internet connectivity, produce relevant mobile systems for communication, disaster mapping and response and guarantee electricity supply.
